# Effects of frankincense on experimentally induced renal stones in rats

**DOI:** 10.1002/bco2.227

**Published:** 2023-02-09

**Authors:** Mohamed S. Al‐Marhoon, Ahmed Al‐Harrasi, Khurram Siddiqui, Mohammed Ashique, Haytham Ali, Badreldin H. Ali

**Affiliations:** ^1^ Urology Division, Department of Surgery, College of Medicine and Health Sciences Sultan Qaboos University Seeb Sultanate of Oman; ^2^ Natural and Medical Sciences Research Center University of Nizwa Nizwa Sultanate of Oman; ^3^ Department of Pharmacology and Clinical Pharmacy, College of Medicine and Health Sciences Sultan Qaboos University Seeb Sultanate of Oman; ^4^ Department of Animal and Veterinary Sciences, College of Agricultural and Marine Sciences Sultan Qaboos University Seeb Sultanate of Oman

**Keywords:** Boswellia, frankincense, kidney, Luban, rats, renal, stones

## Abstract

**Objectives:**

Frankincense (Luban) is a resin obtained from trees of genus *Boswellia*. The south of Oman hosts *Boswellia sacra* trees known to have many social, religious and medicinal uses. The anti‐inflammatory and therapeutic potential of Luban has recently attracted the interest of the scientific community. The aim is to study the efficacy of Luban water extract and its essential oils on experimentally induced renal stones in rats.

**Materials and Methods:**

A rat model of urolithiasis induced by *trans*‐4‐hydroxy‐L‐proline (HLP) was used. Wistar Kyoto rats (27 males, 27 females) were randomly distributed into nine equal groups. Treatment groups were given Uralyt‐U (standard) or Luban (50, 100 and 150 mg/kg/day), starting Day 15 from HLP induction for a duration of 14 days. The prevention groups were given Luban in similar doses, starting Day 1 of HLP induction for 28 days. Several plasma biochemical and histological parameters were recorded. Data were analysed with GraphPad Software. Comparisons were performed by one‐way analysis of variance (ANOVA) and the Bonferroni test.

**Results:**

The lithogenic effects of HLP, such as an increase in urine oxalate and cystine, an increase in plasma uric acid and an increase in kidney levels of calcium and oxalate, have all been best significantly reversed by the Luban dose of 150 mg/kg/day. The histological changes of HLP on the kidney tissue including calcium oxalate crystal formation, cystic dilatation, high degree of tubular necrosis, inflammatory changes, atrophy and fibrosis have also been ameliorated by Luban dose of 150 mg/kg/day.

**Conclusion:**

Luban has shown a significant improvement in the treatment and prevention of experimentally induced renal stones, particularly at a dose of 150 mg/kg/day. Further studies on the effect of Luban in other animal models and humans with urolithiasis are warranted.

## 1 INTRODUCTION

Frankincense or olibanum is the oleogum aromatic resin that is harvested from trees belonging to the genus *Boswellia*. The major sources of commercial frankincense are *B. serrata* (India), *B. sacra* (Oman) and *B. carteri* (Somalia). The gum resin of the *Boswellia trees* is obtained by the incision of the stem or branches. The word frankincense is derived from the ancient French name ‘frankincense’ meaning ‘pure incense’ and is known in Arabic as ‘Luban’. It has been used as incense since ancient times and in recent years in the preparation of cosmetics and perfumes. Pharmacologically, it has been shown to possess anti‐inflammatory, sedative, anti‐hyperlipidaemic, anti‐bacterial and anti‐cancer properties.^1^, ^2^, ^3^

Animal studies and pilot clinical trials support the potential of *B. serrata* gum resin extract (BSE) for the treatment of a variety of inflammatory diseases.^4^ The pharmacological actions of BSE have been mainly attributed to boswellic acids, especially 11‐keto‐β‐boswellic acid (KBA) and acetyl‐11‐keto‐β‐boswellic acid (AKBA), which were proposed to act as selective 5‐lipoxygenase (5‐LO) inhibitors.^5^ The main components are volatile oils (5–15%), pure resin (55–66%), mucus (12–23%) and boswellic acids (30%).^6^, ^7^ Recently, it has been shown that oral administration of the aqueous stem bark extract of *B. papyrifera* has a dose‐ and time‐dependent nephrocurative effects on acetaminophen‐induced kidney damage in rats.^8^ In addition, Oleo‐gum‐resin of *B. serrata* Roxb induced renoprotective action against gentamicin‐induced nephrotoxicity in Albino rats.^9^ Also, *Zingiber officinale* Roscoe (Ginger), gum arabic (AG) and *Boswellia* have been found to be beneficial adjuvant therapy in patients with acute and chronic renal failure to prevent disease progression and delay the need for renal replacement therapy.^10^

## 2 MATERIALS AND METHODS

### 2.1 Animals

Adult male and female Wistar Kyoto (WKY) rats (9–10 weeks old, weighing 240 ± 15 g) were housed in a dedicated room in the small animal house facility of Sultan Qaboos University (SQU). The rats were kept at an ambient temperature of 22 ± 2°C, relative humidity of about 60% and with a 12 h light–dark cycle (lights on at 6:00 a.m.). Clean tap water and standard pellet chow diet containing 0.85% phosphorus, 1.12% calcium, 0.35% magnesium, 25.3% crude protein and 2.5 IU/g vitamin D3 (Oman Flour Mills Co. SAOG, Muscat, Oman) were provided ad libitum.

### 2.2 Experimental design

After an acclimatization period of 1 week, the animals were divided into nine groups of six animals each (three males, three females), and the experiment of males and females was conducted in two batches. Male WKY rats (*n* = 27) and female WKY rats (*n* = 27) were randomly distributed into nine equal groups (with three rats each) and treated for 28 days as follows (Tables 1 and 2): group 1, control (Con), continued to receive tap water and the same diet without any treatment until the end of the study.^11^ Group 2 was given *trans*‐4‐hydroxy‐L‐proline (HLP) mixed with standard feed for 28 days. For this group, HLP (5%^w/w^) was homogenously mixed with the powdered standard pellet diet and given ad libitum. This group is the ‘lithogenic control’ and was denoted as lithogenic (H).^12^, ^13^ Group 3 was given HLP diet as in group 2 for 28 days. Two weeks after the commencement of the HLP diet, this group was given Uralyt‐U (50 mg/kg/day) from Day 15 onward until the end of the treatment period (28 days). This group was denoted as Uralyt‐U (H + U). Therapeutic regimen included; group 4 was given HLP diet as in group 2 for 28 days. Two weeks after the commencement of the HLP diet, this group was given *B. sacra* (50 mg/kg/day) from Day 15 onward until the end of the treatment period (28 days). This group was denoted as (H + B‐50). Group 5 was given HLP diet as in group 2 for 28 days. Two weeks after the commencement of the HLP diet, this group was given *B. sacra* (100 mg/kg/day) from Day 15 onward until the end of the treatment period (28 days). This group was denoted as (H + B‐100). Group 6 was given HLP diet as in group 2 for 28 days. Two weeks after the commencement of the HLP diet, this group was given *B. sacra* (150 mg/kg/day) from Day 15 onward until the end of the treatment period (28 days). This group was denoted as (H + B‐150). Preventive regimen included; group 7 was given HLP diet as in group 2 for 28 days. Additionally, this group was given *B. sacra* (50 mg/kg/day) concomitantly from Day 1 onward until the end of the treatment period (28 days). This group was denoted as p (H + B‐50). Group 8 was given HLP diet as in group 2 for 28 days. Additionally, this group was given *B. sacra* (100 mg/kg/day) concomitantly from Day 1 onward until the end of the treatment period (28 days). This group was denoted as p (H + B‐100). Group 9 was given HLP diet as in group 2 for 28 days. Additionally, this group was given *B. sacra* (150 mg/kg/day) concomitantly from Day 1 onward until the end of the treatment period (28 days). This group was denoted as p (H + B‐150).

**TABLE 1:** Summary of study groups.

Group	Name	Diet	Intervention
1	Control (Con)	Regular rat feed and drinking water	No
2	Lithogenic (H)	Chow mixed with 5% HLP for 28 days	Vehicle (1% Tween 80) for 28 days
3	Uralyt‐U (H + U)	Chow mixed with 5% HLP for 28 days	Uralyt‐U (50 mg/kg/day) for 15–28 days
4	Treatment 1 (H + B‐50)	Chow mixed with 5% HLP for 28 days	*Boswellia sacra* (50 mg/kg/day), from 15 to 28 days
5	Treatment 2 (H + B‐100)	Chow mixed with 5% HLP for 28 days	*B. sacra* (100 mg/kg/day), from 15 to 28 days
6	Treatment 3 (H + B‐150)	Chow mixed with 5% HLP for 28 days	*B. sacra* (150 mg/kg/day), from 15 to 28 days
7	Prevention 1 p (H + B‐50)	Chow mixed with 5% HLP for 28 days	*B. sacra* (50 mg/kg/day), from 1 to 28 days
8	Prevention 2 p (H + B‐100)	Chow mixed with 5% HLP for 28 days	*B. sacra* (100 mg/kg/day), from 1 to 28 days
9	Prevention 3 p (H + B‐150)	Chow mixed with 5% HLP for 28 days	*B. sacra* (150 mg/kg/day), from 1 to 28 days

Abbreviations: HLP, hydroxy‐L‐proline; Luban, frankincense *Boswellia Sacra*.

**TABLE 2:**
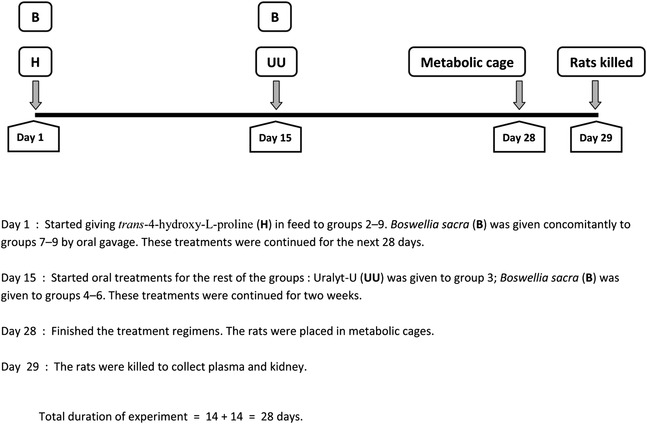
Experiment outline.

The rats were monitored daily till the end of the experiment. They were weighed on a weekly basis during the experimental period. On the final day of treatment (Day 28), the rats were placed individually in metabolic cages for 24 h. The next day, urine voided in the previous 24 h was collected in sterile tubes, and the total volume voided recorded. The pH of urine samples was measured, and the samples were stored at −80°C pending analysis. The feed and water intake and faeces output were also noted. The rats were weighed and anaesthetized with ketamine (75 mg/kg) and xylazine (5 mg/kg) administered intraperitoneally. The rats were carefully dissected to expose the abdominal cavity, and blood (about 6 mL) was collected from the inferior vena cava. The blood collected was centrifuged at 900*g* at 4°C for 15 min to separate plasma. These plasma samples were stored at −80°C until analysis. From each rat, the two kidneys were excised, cleaned by blotting on a filter paper and weighed to record the total kidney weight. A small piece of the right kidney was fixed in formol–saline for subsequent histopathological examinations. The rest of the right kidney and the left kidney were quickly wrapped in aluminium foils and rapidly dipped in liquid nitrogen. These were later transferred to a −80°C deep freezer and stored until analysis. A portion of this frozen kidney tissue was used to prepare 10%^w/v^ renal homogenates in phosphate‐buffered saline (PBS); the supernatant was analysed for calcium, oxalate and phosphorus.^14^

### 2.3 Biochemical measurements

The pH of urine was measured using pH test strips (Macherey‐Nagel GmbH & Co. KG, Düren, Germany). Urine osmolality was measured using a freezing point osmometer (Osmomat 3000, Gonotec GmbH, Berlin, Germany). All of the tests in plasma, urine and renal homogenates were done using standard ELISA or colorimetric assay kits. Biomarkers of renal function (urea and creatinine) and magnesium in plasma and uric acid and phosphorus in both urine and plasma were all measured using an automated machine (Mindray BS‐120 Chemistry Analyzer, Shenzhen Mindray Bio‐Medical Electronics Co., Shenzhen, China). Cysteine, sodium and potassium were measured using colorimetric assay kits from Elabscience (Houston, TX, USA). Calcium, oxalate, citrate and phosphate were estimated using colorimetric assay kits from BioVision (Milpitas, CA, USA).^15^

### 2.4 Histopathology

The kidneys were excised, sliced and then routinely processed for histopathology analyses. The pathologists were blinded to the treatment type received by rats. We made 4 μm sections from each kidney sample and stained them individually with three stains: haematoxylin and eosin (H&E), picro–Sirius red (PSR) and periodic acid–Schiff (PAS). All the three stains were purchased from Abcam (Cambridge, UK). A semi‐quantitative method was used to score renal tubular necrosis on a scale of 0–4 where 0 = normal, no necrosis; 1 < 10%; 2 = 10–25%; 3 = 26–75%; 4 > 75%.^16^ Three 40× fields were assessed from each kidney section of each rat from the nine groups, and the score was calculated according to the mean percentage. Fibrosis index was calculated using PSR staining, and tubular atrophy was assessed using the PAS staining techniques. Polarized microscope filter was used to show the birefringence properties of calcium oxalate and Sirius red‐stained collagen fibres.

Sirius red‐stained slides were analysed following the procedure described by Manni et al.^17^ The slides were examined using Olympus BX51 microscope attached to Olympus DP70 camera, and images were acquired using the 40× objective lens. Three random images of the renal cortex were acquired from each kidney section of each rat from the nine groups and stored in TIFF 24‐bit RGB colour image format. Exact camera and microscope settings were retained unchanged during the imaging process. Image analysis of the stored images was performed using ImageJ® image analysis software (http://rsbweb.nih.gov/ij/). Briefly, the images were transformed into grey scale, and the red‐stained collagen was isolated using the hue histogram filter available in ‘threshold colour’ followed by measuring the isolated area as a percentage. The fibrosis index percentage was calculated to assess the tissues' collagen content by calculating the ratio of the mean Sirius red‐stained positive area to the whole mean area of each photomicrograph (for each rat).

## 3 MATERIALS

### 3.1 Uralyt‐U preparation

Uralyt‐U granules (MEDA Pharma GmbH & Co. KG, Benzstrasse, Germany) were dissolved in distilled water and administered by oral gavage to the rats at a dose of 50 mg/kg/day. The dose of Uralyt‐U was chosen in accordance with previous research.^18^ During the treatment period, special care was taken to keep the pH of the urine of rats in the optimal range of 6.2–6.8 by continuous monitoring.

### 3.2 Preparation of frankincense ( *B. sacra* )

In this study, we used the finest Omani Hojari Frankincense, which is the best in the market of the Sultanate of Oman based on expert opinion.^19^, ^20^ Frankincense was obtained from the frankincense tree (*B. sacra*) growing in the Dhofar region, Sultanate of Oman. An aqueous solution of frankincense resin powder was prepared in distilled water. The dissolution was achieved by sonication and vigorous shaking for an extended period (overnight). The final concentration of frankincense in the solution was 75 mg/mL. This solution was administered to the rats by oral gavage at doses of 50, 100 and 150 mg/kg/day, as mentioned above. The doses of frankincense were selected based on previous studies that investigated the effectiveness of frankincense on cognitive functions in rats.^21^ As there are no previous reports on any effective dose of frankincense on urolithiasis models in our knowledge, we decided to test three graded doses of frankincense—50, 100 and 150 mg/kg in this study.

### 3.3 *trans*‐4‐Hydroxy‐L‐proline

*trans*‐4‐Hydroxy‐L‐proline was purchased from AK Scientific (Union City, CA, USA). The rest of the chemicals and reagents used were also of the highest purity grade.

## 4 STATISTICAL ANALYSIS

Data were expressed as means ± SEM and were analysed with GraphPad Prism version 5.03 for Windows software (GraphPad Software Inc., San Diego, CA, USA). Comparisons between the groups were performed by one‐way analysis of variance (ANOVA), followed by Bonferroni comparisons. *P* values < 0.05 were considered significant.

## 5 RESULTS

The change in body weight (%) from the start to end of the experiment was 25%, (mean ± SEM) 24.2 ± 1.6, 15% (15.12 ± 0.98), 12% (11.76 ± 0.86) and 10% (9.04 ± 0.74) for group 1, control (Con), group 9, p (H + B‐150), group 6, (H + B‐150), and group 2, lithogenic (H), respectively. Rats in control group 1 (Con) had the best change in body weight (25%), whereas the lithogenic control group 2 (H) had the worst change in body weight (10%). The rats who received the dose of 150 mg/kg/day *Boswellia* as treatment (12%) or prevention (15%) had the highest change in body weight (%) than those who received the Boswellia dose of 50 or 100 mg/kg/day. Water intake and urine output and flow have increased in the lithogenic group 2 (H) rats compared with the control group 1 (Con). However, food intake and faeces output have decreased in the lithogenic group 2 (H) rats, with the best increase achieved by group 6, (H + B‐150), and group 3, (H + U).

**FIGURE 1:**
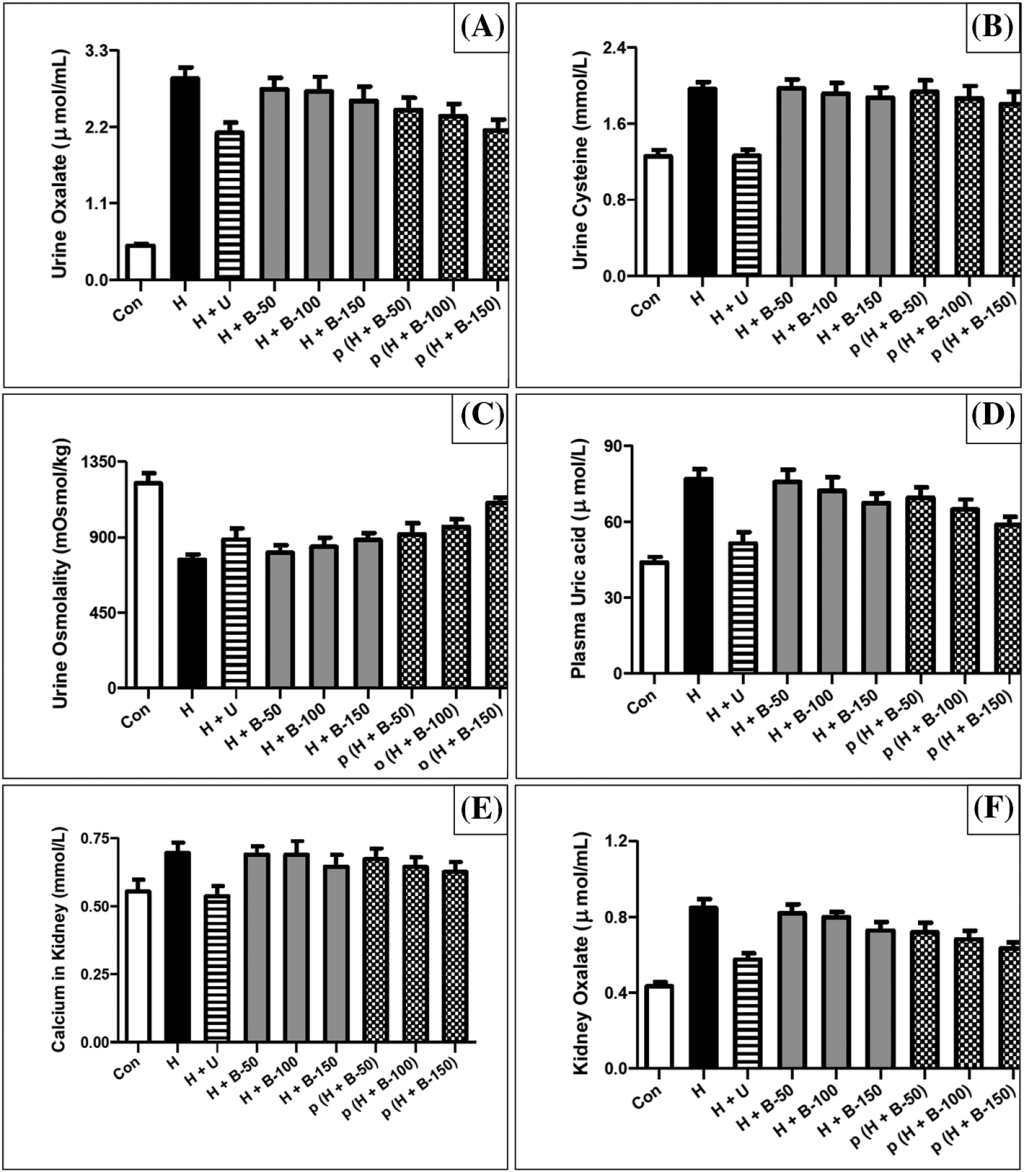
Biochemical and physical changes. (A) oxalate in urine, (B) cysteine in urine, (C) osmolality in urine, (D) uric acid in plasma, (E) calcium in kidney, (F) oxalate in kidney. Group 1: control (Con); group 2: lithogenic (H); group 3: Uralyt‐U (H + U); group 4: (H + B‐50); group 5: (H + B‐100); group 6: (H + B‐150); group 7: p (H + B‐50); group 8: p (H + B‐100); group 9: p (H + B‐150).

The urine biochemical analysis showed that oxalate increased significantly (*P* < 0.0001) in the lithogenic control group 2 (H) compared with the control group 1 (Con). Thereafter, the highest significant (*P* < 0.001) reduction in urine oxalate was achieved in group 3, Uralyt‐U (H + U), group 6, (H + B‐150), and group 9, p (H + B‐150) (Figure 1A). Urine cystine increased significantly (*P* < 0.0001) in the lithogenic control group 2 (H) compared with the control group 1 (Con). Thereafter, the highest significant (*P* < 0.0001) reduction in urine cystine was achieved in group 3, Uralyt‐U (H + U), with non‐significant reduction by group 6, (H + B‐150), and group 9, p (H + B‐150) (Figure 1B). Urine osmolality reduced significantly (*P* < 0.0001) in the lithogenic control group 2 (H) compared with the control group 1 (Con). Thereafter, it improved significantly (*P* < 0.0001) in group 7, p (H + B‐50), group 8, p (H + B‐100), and group 9, p (H + B‐150) (Figure 1C).

The serum biochemical analysis showed that uric acid increased significantly (*P* < 0.0001) in the lithogenic control group 2 (H) compared with the control group 1 (Con). Thereafter, the highest significant (*P* < 0.001) reduction in uric acid was achieved in group 8, p (H + B‐100), and group 9, p (H + B‐150) (Figure 1D). The kidney homogenate biochemical analysis showed that calcium increased significantly (*P* < 0.05) in the lithogenic control group 2 (H) compared with the control group 1 (Con). Thereafter, the highest significant (*P* < 0.001) reduction in calcium was achieved in group 3, Uralyt‐U (H + U), with non‐significant reduction by group 6, (H + B‐150), and group 9, p (H + B‐150) (Figure 1E). Kidney oxalate increased significantly (*P* < 0.0001) in the lithogenic control group 2 (H) compared with the control group 1 (Con). Thereafter, the highest significant (*P* < 0.0001) reduction in oxalate was achieved in group 6, (H + B‐150), and group 9, p (H + B‐150) (Figure 1F).

**FIGURE 2:**
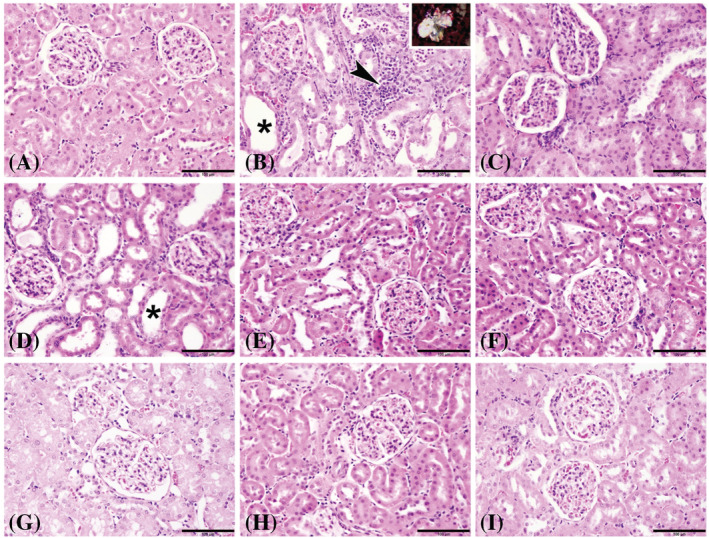
Representative photomicrographs of renal tissues from male rats stained with haematoxylin and eosin where (A) represents control group; (B) group given *trans*‐4‐hydroxy‐L‐proline (HLP) alone; (C) group given HLP and treated with Uralyt‐U (from Day 15 onward); while (D), (E) and (F) represent groups given HLP and then *Boswellia sacra* (from Day 15 onward as treatment) at the doses 50, 100 and 150 mg/kg, respectively; and (G), (H) and (I) represent: groups given HLP and *B. sacra* concomitantly (on all 28 days as a preventive regimen) at the doses 50, 100 and 150 mg/kg, respectively. The asterisks indicate cystic dilatation of renal tubules, and the arrowhead indicates mononuclear cell infiltrations. Inset: intratubular calcium oxalate crystals showing birefringence under polarized light. (Bar = 100 μm).

**FIGURE 3:**
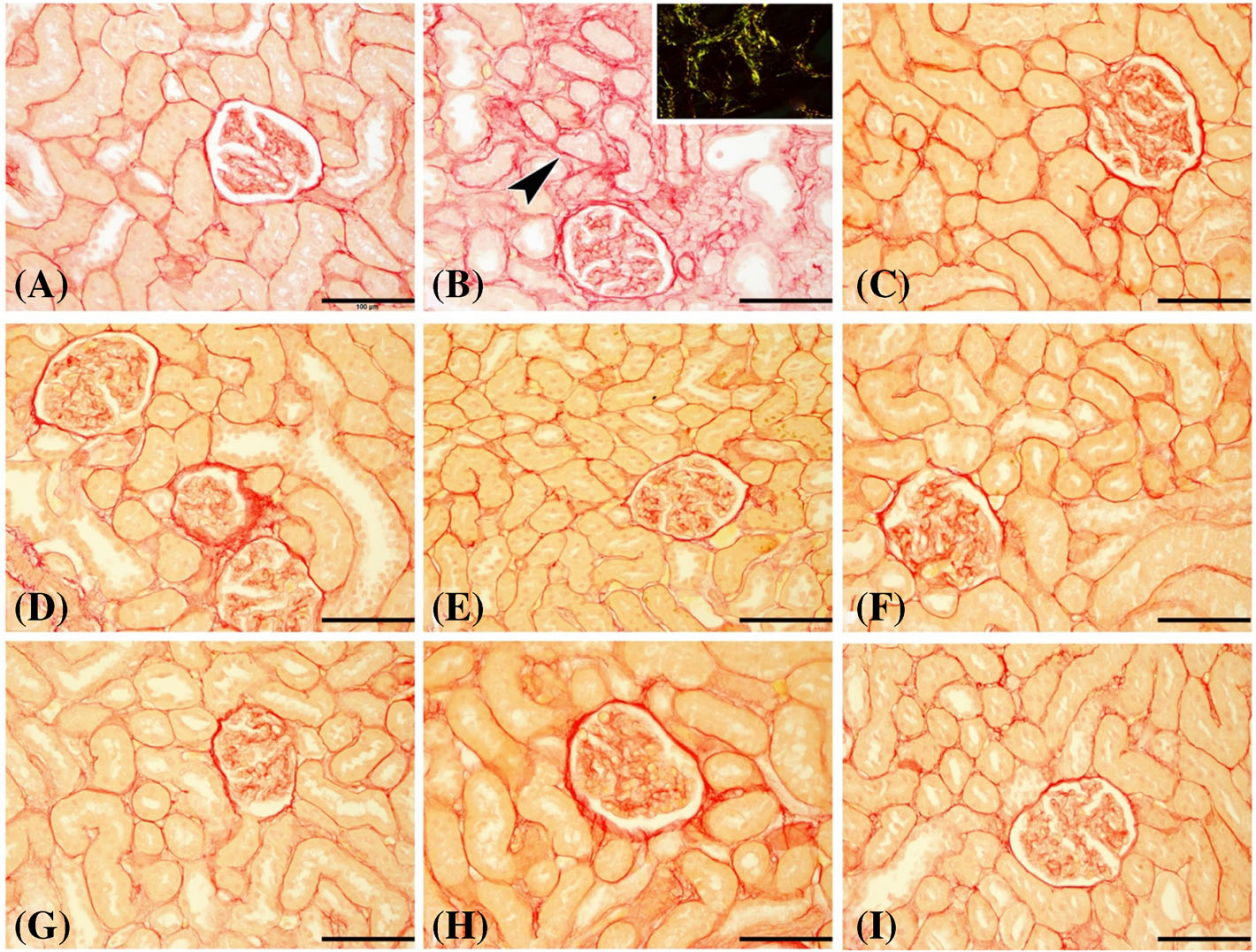
Representative photomicrographs of renal tissues from male rats stained with picro–Sirius red where (A) represents control group; (B) group given *trans*‐4‐hydroxy‐L‐proline (HLP) alone; (C) group given HLP and treated with Uralyt‐U (from Day 15 onward); while (D), (E) and (F) represent groups given HLP and then *Boswellia sacra* (from day 15 onward as treatment) at the doses 50, 100 and 150 mg/kg, respectively; and G, H and I represent: groups given HLP and *B. sacra* concomitantly (on all 28 days as a preventive regimen) at the doses 50, 100 and 150 mg/kg, respectively. The photomicrographs show the distribution of Sirius red‐stained fibrotic areas (arrowhead) and the non‐collagen structures (stained in yellow). *Inset*: birefringence of the stained collagen fibres using polarized light. (Bar = 100 μm).

The histopathology of male rats (batch 1) shows the microscopic examination of the renal tissues from different groups that revealed normal histological structures with intact glomerular tufts and renal tubules (lesion score 0) in group 1, control (Con) (Figure 2A). Examined renal tissues of group 2, lithogenic (H), exhibited cystic dilatation, proteinaceous casts and necrosis of multiple renal tubules (score 4) that were surrounded by mononuclear inflammatory cells, along with the presence of calcium oxalate crystals evidenced by polarized light in few renal tubules (Figure 2B). Group 3, Uralyt‐U (H + U), showed mild basophilia and dilatation of renal tubules with intact glomerular tufts (score 2) (Figure 2C). Renal tissues of group 4, (H + B‐50), showed marked tubular dilatation, tubular protein casts and dilatation of a few renal tubules with intact glomeruli (score 3) (Figure 2D). In group 5, (H + B‐100), group 6, (H + B‐150), and group 7, p (H + B‐50), mild basophilia and dilatation of renal tubules with intact glomerular tufts (score 2) were observed (Figure 2E–G, respectively). Examined tissues from group 5, (H + B‐100), and group 9, p (H + B‐150), showed mild dilatation of renal tubules with intact glomeruli (score 1) (Figure 2H,I). The lesion scores for each group are shown in Table 3. The distribution of collagen fibres (stained in red) and the non‐collagen structures (stained in yellow) was demonstrated using PSR stain in all the nine groups (Figure 3A–I). Also, the fibrosis index percentage was calculated and it was higher in group 2, lithogenic (H), 35.4% and group 4, (H + B‐50), 22.3%. Meanwhile, tubular atrophy was evidenced in HLP‐treated groups by the thickening and corrugation of the basement membrane of renal tubules and the loss of the brush borders, as shown by PAS staining in all nine groups. However, tubular atrophy was predominantly found in group 2, lithogenic (H), and to a lesser extent in group 4, (H + B‐50).

**TABLE 3:** Histopathological characteristics‐tubular necrosis and fibrosis index‐in renal tissues of rats from all the nine experimental groups: group 1: control (Con); group 2: lithogenic (H); group 3: Uralyt‐U (H + U); group 4: (H + B‐50); group 5: (H + B‐100); group 6: (H + B‐150); group 7: p (H + B‐50); group 8: p (H + B‐100); group 9: p (H + B‐150).

	Tubular necrosis			Fibrosis index %		
	Lesion score					
Group	Male	Female	Average	Male	Female	Average
Con	0	0	0	6.3	5.4	5.85
H	4	4	4	35.4	28.3	31.85
H + U	2	2	2	8.4	11.3	9.85
H + B‐50	3	3	3	22.3	33.4	27.85
H + B‐100	2	2	2	7.2	11.4	9.3
H + B‐150	2	2	2	8.2	10.2	9.2
p (H + B‐50)	2	2	2	7.4	8.4	7.9
p (H + B‐100)	1	1	1	9.3	7.1	8.2
p (H + B‐150)	1	1	1	7.2	7.2	7.2

The histopathology of female rats (batch 2) shows microscopic examination of the renal tissues from different groups revealed normal architecture and structures of the glomeruli and renal tubules (lesion score 0) in group 1, control (Con). Examined renal tissues of group 2, lithogenic (H), showed marked infiltration of the interstitial tissues with mononuclear inflammatory cells with cystic dilatation and necrosis of renal tubules (score 4), and the presence of calcium oxalate crystals in renal tubules was evidenced by polarized light. Group 3, Uralyt‐U (H + U), showed slight tubular dilatation (score 2). Renal tissues of group 4, (H + B‐50), showed interstitial tissue infiltration with mononuclear inflammatory cells, marked tubular dilatation, tubular protein casts and cystic dilatation of renal tubules with intact glomeruli (score 3). Group 5, (H + B‐100), group 6, (H + B‐150), and group 7, p (H + B‐50), exhibited basophilia and dilatation of a few renal tubules with intact glomerular tufts (score 2), respectively. Examined tissues from group 5, (H + B‐100), and group 6, (H + B‐150), showed mild dilatation of renal tubules with intact glomeruli (score 1). The lesion scores for each group are shown in Table 3. The distribution of collagen fibres (stained in red) and the non‐collagen structures (stained in yellow) was demonstrated using PSR stain in all nine Groups. Also, the fibrosis index percentage was calculated (Table 3), and it was higher in group 2, lithogenic (H), 28.3% and in group 4, (H + B50), 33.4%. Meanwhile, tubular atrophy was evidenced in the HLP‐treated groups by the thickening and corrugation of the basement membrane of renal tubules and the loss of the brush borders, as shown by PAS staining in all the nine groups. However, tubular atrophy was predominantly found in group 2, lithogenic (H), and to a lesser extent in group 4, (H + B‐50), and group 8, p (H + B‐100).

## 6 DISCUSSION

In this animal study of a rat model of urolithiasis, the aim was to ascertain the efficacy of *B. sacra* (Luban) in the treatment and prevention of renal stones. The study was carried out on a total of 54 rats (27 males, 27 females) investigating the biochemical and histological changes in the rats' plasma, urine and kidney homogenate. The study included nine groups (Control, lithogenic, Uralyte‐U, three treatment doses and three preventive doses of *B. sacra)*. In this study, we have shown that the lithogenic substance of hydroxy‐L‐proline (HLP) had significant changes in the rats' body weight and behaviour of food and water intake. While the weight was reduced, the feeding and water intake have increased. These effects have been effectively reversed by giving Luban as treatment or prevention. The lithogenic effects of HLP, such as an increase in urine oxalate and cystine, an increase in plasma uric acid and an increase in the kidney levels of calcium and oxalate, have all been best reversed by the Luban dose of 150 mg/kg/day.

The above findings have been proved histologically by looking at kidney tissue effects through quantifying renal tubular necrosis, fibrosis index, tubular atrophy, collagen fibres and the presence of birefringence properties of calcium oxalate. In male and female rats, the lithogenic substance of HLP induced significant changes in the rats including calcium oxalate (CaOx) crystal formation, cystic dilatation, high degree of tubular necrosis (scale 4) with inflammatory changes atrophy and fibrosis. These changes were reversed by Luban treatment of 100 and 150 mg/kg/day and were prevented with Luban 100 and 150 mg/kg/day. Using the Luban dose of 150 mg/kg/day showed the best effects for treatment as the average score for necrosis (scale 2) and fibrosis index (9.2%) were the lowest compared with other groups and in reference to group 2, lithogenic (H), and group 4, (H + B‐50), which had the highest necrosis (scale 3) and fibrosis index (27.85%). In addition, for prevention, using the Luban dose of 150 mg/kg/day showed the best effects as the average score for necrosis (scale 1) and fibrosis index (7.2%) were the lowest compared with other groups and in reference to the group 2, lithogenic (H), and group 7, p (H + B‐50), which had the highest necrosis (scale 3) and fibrosis index (27.85%). In addition, tubular atrophy was highest seen in group 2, lithogenic (H), and treatment or prevention with Luban 50 mg/kg/day, indicating that the dose of 50 mg/kg/day of Luban is not as effective. On the other hand, the histological changes seen in Luban 150 mg/kg/day as treatment or prevention were closer to the normal histology (control) compared with the HLP‐induced histology indicating the powerful effects of Luban 150 mg/kg/day in treatment and preventing the deleterious effects associated with stone formation. No stone crystals (CaOx) were demonstrated in groups treated with Luban or Uralyte‐U. However, Luban in the doses of 100 and 150 mg/kg/day was more effective in reducing the changes of fibrosis induced by HLP than Uralyte‐U, indicating that Luban is more effective than Uralyte‐U. It should be noted that the administration of the Luban extract used in this work did not cause any overt adverse effects on the treated rats, suggesting that the preparations used were safe. However, further detailed studies on the safety of the product on all the major systems of the body are warranted.

The limitations of this study include: limited number of animals in each group to illustrate more histological findings and the need to use electron microscope to show the details of the stone crystals and the features like necrosis and basement membrane thinking; the need to use more robust analysis utilizing molecular techniques in addition to histological and biochemical analysis; the need to isolate the frankincense essential oils and use them separately to see if they have more powerful effects than the water extract of frankincense, which may have lost the oils during the extraction process.

## 7 CONCLUSIONS

This is the first study to investigate the effect of Luban in the treatment and prevention of renal stones. This study showed a good effect of Luban in the treatment and prevention of renal stones, especially at a dose of 150 mg/kg/day. These encouraging results should be confirmed in other animal models of urolithiasis, followed up by clinical trials in humans to further study the effect of Luban on renal stones.

## AUTHOR CONTRIBUTIONS

Mohamed S. Al‐Marhoon is the principal investigator, Ahmed Al‐Harrasi is the collaborating investigator, Khurram Siddiqui participated in the review of the article, Mohammed Ashique is the research assistant, Haytham Ali done the histopathology preparation and studies, and Badreldin H. Ali participated in the supervision of the chemical products used and review of the article.

## CONFLICT OF INTEREST STATEMENT

All authors report no conflict of interest.

## DISCLOSURE OF INTEREST

We disclose that there are no financial or non‐financial interests that are directly or indirectly related to the submission of this publication.
